# Tooth Brush Exercise

**Published:** 1919-09

**Authors:** Sidney Rauh


### TOOTH BRUSH EXERCISE

BY SIDNEY RAUH, D.D.S.

The tooth brush is only partially a cure; its use is primarily a preventive measure. The late Dr. M. H. Fletcher, who did much to bring about this oral hygiene movement, advocated a horizontal stroke with a large straight, stiff brush. Dr. Barnes, of Cleveland, uses the smallest of brushes, as soft as possible, soaked in soap and water. There are numerous other methods, all, however, advocating reaching the gingival margin. This is real standardizing. The essayist shows pictures with the bristles between the teeth. This we have not been able to accomplish. In fact, in my lectures to the public I demonstrate that you can not do this. Therefore, the other appliances, such as the toothpick, floss silk, pastes, washes, etc., and the individual idea of the operator must come into play. There are two distinct problems: one is the problem in your office which you have the privilege of treating just as you would any office problem; the other is the method which the profession must teach to the people to let them know how to keep their mouths clean, and this must be standardized. The personality back of the essayist gives the remarkable results of which he speaks. In our offices, by direct contact, we do get results. But there should be a method whereby a group of twenty-five children can be taught, in a few minutes, how to brush teeth.

Another very important point to my mind is the education of the dentist himself. In this room probably each person is in sympathy—but where is your attendance? How many men attend these meetings regularly? Therefore, your propaganda must extend to the profession before it extends to the public and the individual. We have standardized one thing—the fact that you must reach the gingival margin. If a committee is formed, you can not work out a system in a year. I think it would be a mistake to come before the National Dental Assocaton next year and say, "Here is a system which will work."

We have carried on an oral hygiene movement in Cincinnati for eight years and we have succeeded in standardizing a tooth brush method for the public if not for private operators. When we go to the schools or Mothers' Clubs we have a standardized method. Unfortunately this is a method that the essayist does not advocate, but we do get results. The written instructions are as follows:

TOOTH BRUSH DRILL

*Attention*—Hold brush up in right hand at the heighth of the shoulder, in front of child. (This is to see that the tooth brush is clean.)

*Upper Teeth.* 1. Place brush in mouth, upper right, where the gum and the teeth join. Turn down towards grinding surface eight times.

2. Brush upper front. Place on gum and turn down eight times.

3. Brush upper left. Place brush on gum, turn down eight times.

4. Brush upper right inside. Place brush on gum and turn down eight times.

5. Brush upper front inside. Place brush vertically, brush down eight times.

6. Brush upper left side. Place brush on gum and turn down eight times.

7. Place brush on grinding surface of teeth, upper right, scrub forward and back eight times.

8. Place brush on grinding surface of teeth, upper left, scrub forward and back eight times.

*Lower Teeth.* 9. Place brush in mouth, lower right, where the gum and the teeth join. Turn up towards grinding surface eight times.

10. Brush lower front. Place on gum, turn up eight times.

11. Brush lower left. Place brush on gum, turn up eight times.

12. Brush lower right inside. Place brush on gum, brush up eight times.

13. Brush lower front inside. Place brush vertically, brush up eight times.

14. Brush lower left inside. Place brush on gum, turn up eight times.

15. Place brush on grinding surface of teeth, lower right, scrub forward and back eight times.

16. Place brush on grinding surface of teeth, lower left, scrub forward and back eight times.

*Attention*—Hold brush up in right hand, at the heighth of the shoulder, in front of the child. (See how clean the brush is now.)

Next—See that each child's brush is thoroughly rinsed with fresh water and put away in a clean place.

One time it became necessary for us to raise funds for our work. In that campaign we did various things. Among other schemes, we had to tooth brush drill for publicity. Ten children from one of the schools in a poor district were taught a tooth brush drill in five minutes and placed in a conspicuous show window; they attracted a large crowd, which they held.—*Journal N. D. A.*, April. 1919.

